# Supplementary material for: Multicenter comparative analysis of local and aggregated data training strategies in COVID-19 outcome prediction with Machine learning
Source: PLOS Digit Health. 2024 Dec 26;3(12):e0000699. doi: 10.1371/journal.pdig.0000699 (PMC11670925; doi:10.1371/journal.pdig.0000699)
Supplement: S1 Table — (DOCX) [file pdig.0000699.s001.docx]

**S1 Table.** Descriptive measures (mean, standard deviation and median) of predictors for each hospital.

| **Hospital** | **Descriptive Measures** | **age** | **heart_rate** | **resp_rate** | **sys_press** | **dias_press** | **mean_press** | **temp** | **hemoglobin** | **platelets** | **hematocrit** | **red_cells_count** |
| --- | --- | --- | --- | --- | --- | --- | --- | --- | --- | --- | --- | --- |
| SouthEast - 2 | mean | 59.31 | 87.09 | 24.48 | 119.40 | 71.70 | 87.55 | 36.37 | 12.34 | 234923.74 | 37.68 | NaN |
| SouthEast - 2 | std | 16.00 | 18.50 | 7.38 | 22.56 | 14.46 | 15.57 | 0.89 | 2.54 | 104514.48 | 8.06 | NaN |
| SouthEast - 2 | median | 61.00 | 86.00 | 23.00 | 120.00 | 70.00 | 86.98 | 36.40 | 13.00 | 217000.00 | 39.00 | NaN |
| SouthEast - 3 | mean | 66.17 | 83.75 | 20.26 | 130.75 | 77.16 | 95.53 | 36.34 | 12.74 | 209400.00 | 30.93 | 4.26 |
| SouthEast - 3 | std | 17.04 | 15.18 | 4.62 | 20.56 | 13.04 | 13.93 | 1.17 | 2.03 | 90804.79 | 7.62 | 0.68 |
| SouthEast - 3 | median | 68.00 | 82.00 | 20.00 | 130.00 | 77.00 | 95.00 | 36.40 | 12.90 | 198000.00 | 30.30 | 4.31 |
| SouthEast - 5 | mean | 53.31 | 83.62 | 20.12 | 130.25 | 81.89 | 92.44 | 36.57 | 13.05 | 233661.16 | NaN | 4.51 |
| SouthEast - 5 | std | 15.53 | 12.41 | 2.99 | 15.64 | 10.89 | 12.48 | 0.74 | 2.69 | 83439.95 | NaN | 1.10 |
| SouthEast - 5 | median | 50.50 | 82.00 | 19.50 | 130.00 | 80.00 | 93.50 | 36.40 | 13.55 | 224000.00 | NaN | 4.57 |
| SouthEast - 6 | mean | 66.97 | 90.04 | 20.12 | 122.72 | 74.46 | 87.25 | 36.72 | 11.02 | 201.11 | 33.78 | 3.88 |
| SouthEast - 6 | std | 17.65 | 19.00 | 4.82 | 29.28 | 15.71 | 19.62 | 1.37 | 3.02 | 104.43 | 7.97 | 0.98 |
| SouthEast - 6 | median | 70.50 | 88.00 | 20.00 | 123.50 | 74.00 | 82.00 | 36.60 | 11.70 | 176.50 | 35.60 | 4.13 |
| NorthEast - 1 | mean | 49.42 | 86.48 | 20.19 | 127.99 | 76.78 | 93.56 | 36.36 | 12.91 | 233440.73 | 39.60 | 4.51 |
| NorthEast - 1 | std | 18.20 | 12.30 | 2.61 | 15.69 | 8.49 | 9.18 | 0.66 | 1.97 | 89291.78 | 5.09 | 0.65 |
| NorthEast - 1 | median | 47.00 | 86.00 | 20.00 | 125.25 | 79.50 | 93.00 | 36.30 | 13.10 | 219000.00 | 40.10 | 4.58 |
| NorthEast - 2 | mean | 62.60 | 81.08 | 18.72 | 120.93 | 72.74 | 88.24 | 36.29 | 12.77 | 449322.92 | 38.53 | 2.39 |
| NorthEast - 2 | std | 17.71 | 18.56 | 5.07 | 27.39 | 18.08 | 20.88 | 1.33 | 1.73 | 64176.28 | 4.78 | 0.99 |
| NorthEast - 2 | median | 65.00 | 80.00 | 18.00 | 120.00 | 77.50 | 91.31 | 36.20 | 12.80 | 451500.00 | 38.60 | 2.22 |
| NorthEast - 3 | mean | 56.99 | 90.89 | 22.74 | 126.55 | 78.12 | NaN | 37.33 | 11.11 | 250437.51 | 34.75 | 3.85 |
| NorthEast - 3 | std | 17.36 | 15.17 | 5.34 | 21.01 | 15.62 | NaN | 0.98 | 2.58 | 118927.82 | 7.33 | 0.63 |
| NorthEast - 3 | median | 58.00 | 90.00 | 22.00 | 120.00 | 80.00 | NaN | 37.20 | 11.20 | 246000.00 | 35.50 | 3.70 |
| NorthEast - 4 | mean | 56.15 | 86.00 | 19.60 | 120.40 | 72.44 | 88.43 | 36.04 | 9.99 | 188963.47 | 30.39 | 3.52 |
| NorthEast - 4 | std | 16.14 | 18.88 | 4.00 | 25.16 | 16.33 | 18.39 | 0.93 | 2.65 | 145655.01 | 7.71 | 0.89 |
| NorthEast - 4 | median | 58.00 | 87.00 | 19.00 | 117.00 | 71.00 | 87.00 | 36.00 | 9.80 | 157050.00 | 30.25 | 3.61 |
| MidWest -1 | mean | 57.99 | 88.27 | 24.39 | 128.20 | 76.82 | 93.26 | 36.85 | 13.17 | 241.07 | 40.23 | 4.39 |
| MidWest -1 | std | 15.78 | 21.06 | 8.16 | 24.79 | 15.81 | 17.21 | 14.80 | 2.39 | 95.07 | 7.70 | 0.72 |
| MidWest -1 | median | 57.00 | 87.00 | 22.00 | 128.00 | 78.00 | 94.00 | 36.20 | 13.40 | 231.50 | 41.00 | 4.47 |
| South - 1 | mean | 60.11 | 86.78 | 19.94 | 131.19 | 74.28 | 13.29 | 36.17 | 13.40 | 196635.79 | 39.26 | NaN |
| South - 1 | std | 17.93 | 20.34 | 3.71 | 20.04 | 11.93 | 33.09 | 5.14 | 1.93 | 83172.26 | 5.06 | NaN |
| South - 1 | median | 60.00 | 84.50 | 20.00 | 125.50 | 74.00 | 0.00 | 36.80 | 13.60 | 182500.00 | 39.80 | NaN |
| South - 2 | mean | 49.62 | 87.06 | 19.05 | 126.11 | 78.12 | 94.12 | 36.76 | 13.55 | 207166.89 | 40.91 | 4.73 |
| South - 2 | std | 17.79 | 14.19 | 2.22 | 20.95 | 12.39 | 14.16 | 0.83 | 1.52 | 63811.03 | 5.33 | 0.97 |
| South - 2 | median | 47.50 | 87.00 | 18.00 | 124.00 | 77.00 | 93.00 | 36.60 | 13.70 | 202000.00 | 41.50 | 4.65 |
| South - 3 | mean | 60.66 | 83.72 | 21.85 | 128.15 | 76.14 | 105.67 | 36.52 | 12.19 | 218055.56 | 36.58 | NaN |
| South - 3 | std | 16.06 | 17.08 | 4.70 | 21.21 | 14.82 | 10.21 | 1.25 | 2.48 | 99171.44 | 7.08 | NaN |
| South - 3 | median | 65.00 | 82.00 | 21.00 | 124.00 | 76.50 | 110.00 | 36.30 | 12.60 | 196500.00 | 37.70 | NaN |
| North - 1 | mean | 56.63 | 82.21 | 21.01 | 123.53 | 76.55 | 79.27 | 36.27 | 12.17 | 285958.12 | 36.95 | 4.30 |
| North - 1 | std | 17.07 | 15.32 | 3.64 | 14.27 | 10.96 | 32.43 | 0.63 | 1.77 | 113679.09 | 4.81 | 0.60 |
| North - 1 | median | 56.00 | 80.00 | 20.00 | 120.00 | 80.00 | 90.00 | 36.10 | 12.40 | 264000.00 | 37.35 | 4.37 |
| North - 2 | mean | 57.15 | 91.22 | 24.46 | 133.32 | 77.93 | 94.71 | 36.47 | 10.76 | 257936.44 | 33.48 | 3.92 |
| North - 2 | std | 15.61 | 21.15 | 8.05 | 24.34 | 14.79 | 14.42 | 0.91 | 2.90 | 148993.42 | 7.75 | 0.87 |
| North - 2 | median | 61.00 | 89.00 | 23.00 | 130.00 | 76.00 | 96.15 | 36.50 | 11.40 | 223900.00 | 34.91 | 4.05 |

**S1 Table.** Descriptive measures (mean, standard deviation and median) of predictors for each hospital.

| **Hospital** | **Descriptive Measures** | **hcm** | **rdw** | **mcv** | **leukocytes** | **neutrophil** | **lymphocytes** | **basophils** | **eosinophils** | **monocytes** | **crp** |
| --- | --- | --- | --- | --- | --- | --- | --- | --- | --- | --- | --- |
| SouthEast - 2 | mean | 28.84 | 14.22 | 85.80 | 9782.92 | 7977.89 | 997.98 | 0.02 | NaN | 0.51 | 1.55 |
| SouthEast - 2 | std | 2.38 | 2.17 | 6.31 | 10866.33 | 4711.53 | 1314.46 | 0.08 | NaN | 0.45 | 1.13 |
| SouthEast - 2 | median | 29.00 | 13.70 | 85.80 | 8555.00 | 7060.00 | 840.00 | 0.01 | NaN | 0.42 | 1.27 |
| SouthEast - 3 | mean | 30.02 | 12.78 | 91.61 | 7633.10 | 5497.60 | 1245.80 | 0.01 | 0.08 | 438.21 | 14.41 |
| SouthEast - 3 | std | 2.13 | 1.75 | 5.50 | 4715.66 | 3794.41 | 716.45 | 0.02 | 0.12 | 272.84 | 11.92 |
| SouthEast - 3 | median | 30.25 | 12.40 | 91.65 | 6400.00 | 4415.00 | 1052.50 | 0.00 | 0.06 | 380.00 | 12.90 |
| SouthEast - 5 | mean | 30.95 | 15.00 | 83.50 | 152.02 | NaN | 211.83 | 17.75 | 114.68 | 428.74 | 64.46 |
| SouthEast - 5 | std | 7.97 | 9.91 | 15.58 | 1210.64 | NaN | 335.63 | 18.14 | 165.56 | 287.28 | 73.35 |
| SouthEast - 5 | median | 30.30 | 13.35 | 87.30 | 8.27 | NaN | 2.05 | 10.00 | 80.00 | 400.00 | 37.25 |
| SouthEast - 6 | mean | 29.06 | 14.61 | 87.72 | 10.93 | 8.34 | 4.97 | NaN | 0.21 | 54.30 | 52.96 |
| SouthEast - 6 | std | 2.97 | 2.28 | 7.76 | 6.50 | 4.42 | 9.58 | NaN | 0.95 | 400.80 | 32.65 |
| SouthEast - 6 | median | 29.76 | 14.20 | 88.36 | 10.32 | 8.20 | 1.19 | NaN | 0.00 | 0.56 | 64.35 |
| NorthEast - 1 | mean | 29.06 | 13.61 | 88.50 | 7196.14 | 4951.30 | 1389.87 | 13.81 | 75.19 | 609.55 | 80.85 |
| NorthEast - 1 | std | 2.18 | 1.81 | 6.24 | 5669.55 | 3270.25 | 793.39 | 18.54 | 128.95 | 375.46 | 96.90 |
| NorthEast - 1 | median | 29.25 | 13.20 | 88.70 | 6320.00 | 4020.00 | 1259.00 | 10.00 | 31.00 | 558.00 | 40.30 |
| NorthEast - 2 | mean | 28.52 | 13.98 | 86.15 | 7750.29 | 3611.71 | 957.81 | 3.58 | 40.95 | 439.37 | 10.46 |
| NorthEast - 2 | std | 1.94 | 1.40 | 5.57 | 8167.18 | 1961.76 | 570.13 | 8.98 | 79.27 | 240.22 | 8.38 |
| NorthEast - 2 | median | 28.60 | 13.70 | 86.30 | 6910.00 | 3725.00 | 903.00 | 0.00 | 0.00 | 427.50 | 8.41 |
| NorthEast - 3 | mean | 29.63 | 14.17 | 89.38 | 10483.12 | 5785.09 | 1140.92 | 19.05 | 190.09 | 577.77 | 19.98 |
| NorthEast - 3 | std | 2.91 | 2.21 | 9.47 | 8190.87 | 3931.89 | 1076.51 | 37.99 | 634.71 | 395.95 | 35.55 |
| NorthEast - 3 | median | 29.80 | 13.20 | 89.55 | 9170.00 | 5343.00 | 810.00 | 2.00 | 60.00 | 567.00 | 12.00 |
| NorthEast - 4 | mean | 28.54 | 14.76 | 86.66 | 11913.29 | 7142.40 | 1237.11 | 31.97 | 140.46 | 478.80 | 7.15 |
| NorthEast - 4 | std | 3.03 | 2.83 | 7.59 | 25247.81 | 7073.95 | 827.33 | 38.09 | 243.91 | 319.12 | 5.54 |
| NorthEast - 4 | median | 28.80 | 13.90 | 86.45 | 7892.50 | 5487.50 | 990.00 | 22.00 | 39.00 | 453.00 | 5.58 |
| MidWest -1 | mean | 30.04 | 13.65 | 91.63 | 11.00 | 2241.51 | 157.52 | 3.19 | 5.01 | 210.10 | 138.23 |
| MidWest -1 | std | 4.44 | 1.93 | 12.25 | 8.61 | 4074.25 | 398.44 | 18.62 | 28.50 | 411.06 | 108.43 |
| MidWest -1 | median | 30.16 | 13.60 | 91.80 | 9.80 | 77.00 | 12.50 | 0.00 | 0.00 | 9.85 | 108.20 |
| South - 1 | mean | 30.50 | 13.67 | 88.42 | 7471.90 | 5045.57 | 1191.51 | 16.64 | 35.19 | 607.62 | 6.66 |
| South - 1 | std | 1.53 | 1.01 | 4.10 | 13590.28 | 3300.37 | 811.23 | 18.33 | 67.66 | 364.06 | 7.07 |
| South - 1 | median | 30.60 | 13.50 | 88.80 | 5955.00 | 4120.00 | 1035.00 | 10.00 | 10.00 | 550.00 | 3.89 |
| South - 2 | mean | 29.13 | 13.55 | 87.67 | 5524.98 | 3793.95 | 1300.00 | 36.29 | 61.67 | 546.47 | 39.96 |
| South - 2 | std | 1.96 | 1.67 | 5.80 | 2794.23 | 2131.79 | 665.03 | 38.28 | 103.63 | 289.83 | 52.51 |
| South - 2 | median | 29.25 | 13.40 | 88.35 | 5250.00 | 3275.00 | 1182.50 | 26.75 | 33.90 | 489.60 | 18.02 |
| South - 3 | mean | 29.14 | 13.69 | 87.72 | 1728.92 | NaN | 1411.36 | 0.02 | NaN | 448.32 | 128.68 |
| South - 3 | std | 2.30 | 2.13 | 6.07 | 4656.03 | NaN | 1486.29 | 0.15 | NaN | 228.19 | 98.94 |
| South - 3 | median | 29.35 | 13.20 | 86.55 | 930.00 | NaN | 1050.00 | 0.00 | NaN | 400.00 | 109.25 |
| North - 1 | mean | 27.68 | 13.64 | 86.22 | 4843.38 | 7726.53 | 1459.18 | 0.44 | 1.57 | 652.75 | 8.99 |
| North - 1 | std | 3.94 | 1.45 | 5.69 | 4678.69 | 1003.23 | 879.14 | 0.53 | 1.18 | 229.50 | 9.28 |
| North - 1 | median | 28.36 | 13.20 | 86.16 | 3960.00 | 7900.00 | 1200.00 | 0.00 | 1.00 | 600.00 | 5.90 |
| North - 2 | mean | 27.86 | 13.09 | 85.07 | 12467.83 | 10782.23 | 938.84 | 55.21 | 72.84 | 654.18 | 124.64 |
| North - 2 | std | 2.01 | 2.18 | 5.50 | 6138.18 | 6054.57 | 644.63 | 42.40 | 137.86 | 367.24 | 90.97 |
| North - 2 | median | 28.20 | 12.27 | 85.43 | 11640.00 | 9897.50 | 759.00 | 48.50 | 12.00 | 638.50 | 127.00 |
