# Supplementary material for: Multicenter comparative analysis of local and aggregated data training strategies in COVID-19 outcome prediction with Machine learning
Source: PLOS Digit Health. 2024 Dec 26;3(12):e0000699. doi: 10.1371/journal.pdig.0000699 (PMC11670925; doi:10.1371/journal.pdig.0000699)
Supplement: S3 Table — In green, the highest AUROC among the eight strategies for each hospital. Abbreviations: xgb: XGBoost, lgb: LightGBM, and cat: Catboost. (DOCX) [file pdig.0000699.s003.docx]

**S3 Table.** The AUROC achieved with the best algorithm (in parenthesis) in the test data set of each hospital for the eight aggregation strategies concerning the outcome outcome MV use. In green, the highest AUROC among the eight strategies for each hospital. Abbreviations: xgb: XGBoost, lgb: LightGBM, and cat: Catboost.

|  | **Training Strategy** | | | | | | | |
| --- | --- | --- | --- | --- | --- | --- | --- | --- |
| **Hospital** | **Strategy 1** | **Strategy 2** | **Strategy 3** | **Strategy 4** | **Strategy 5** | **Strategy 6** | **Strategy 7** | **Strategy 8** |
| SouthEast2 | 0.754 (lgb) | 0.684 (lgb) | 0.674 (lgb) | 0.638 (cat) | 0.690 (lgb) | 0.665 (cat) | 0.601 (cat) | 0.613 (cat) |
| SouthEast3 | 0.865 (lgb) | 0.685 (lgb) | 0.698 (lgb) | 0.741 (cat) | 0.750 (lgb) | 0.579 (xgb) | 0.539 (xgb) | 0.625 (cat) |
| SouthEast5 | 0.906 (xgb) | 0.688 (lgb) | 0.766 (lgb) | 0.859 (cat) | 0.760 (lgb) | 0.328 (xgb) | 0.615 (lgb) | 0.615 (lgb) |
| SouthEast6 | 0.885 (xgb) | 0.673 (lgb) | 0.635 (cat) | 0.654 (cat) | 0.731 (lgb) | 0.808 (xgb) | 0.885 (xgb) | 0.654 (cat) |
| NorthEast1 | 0.947 (lgb) | 0.666 (lgb) | 0.912 (cat) | 0.867 (lgb) | 0.908 (lgb) | 0.850 (xgb) | 0.824 (lgb) | 0.818 (xgb) |
| NorthEast2 | 0.781 (lgb) | 0.737 (lgb) | 0.735 (xgb) | 0.732 (lgb) | 0.763 (lgb) | 0.668 (cat) | 0.634 (lgb) | 0.672 (lgb) |
| NorthEast3 | 0.779 (xgb) | 0.677 (lgb) | 0.561 (lgb) | 0.691 (lgb) | 0.677 (lgb) | 0.632 (cat) | 0.575 (cat) | 0.754 (cat) |
| NorthEast4 | 0.533 (xgb) | 0.817 (lgb) | 0.735 (xgb) | 0.808 (lgb) | 0.800 (lgb) | 0.667 (xgb) | 0.908 (xgb) | 0.700 (lgb) |
| MidWest1 | 0.617 (xgb) | 0.624 (xgb) | ─ | 0.624 (xgb) | 0.580 (lgb) | 0.446 (xgb) | ─ | 0.446 (xgb) |
| South1 | 0.869 (xgb) | 0.802 (lgb) | 0.812 (lgb) | 0.766 (lgb) | 0.800 (lgb) | 0.656 (cat) | 0.792 (xgb) | 0.779 (lgb) |
| South2 | 0.976 (xgb) | 0.817 (lgb) | 0.905 (xgb) | 0.746 (lgb) | 0.841 (lgb) | 0.802 (xgb) | 0.802 (xgb) | 0.635 (lgb) |
| South3 | 0.567 (cat) | 0.722 (lgb) | 0.556 (xgb) | 0.700 (lgb) | 0.683 (lgb) | 0.683 (lgb) | 0.481 (xgb) | 0.606 (cat) |
| North1 | 0.885 (cat) | 0.689 (lgb) | 0.714 (xgb) | 0.697 (lgb) | 0.681 (lgb) | 0.737 (xgb) | 0.801 (cat) | 0.614 (cat) |
| North2 | 0.648 (xgb) | 0.796 (lgb) | 0.685 (cat) | 0.722 (lgb) | 0.778 (lgb) | 0.667 (lgb) | 0.500 (cat) | 0.546 (xgb) |
