# Supplementary material for: Multicenter comparative analysis of local and aggregated data training strategies in COVID-19 outcome prediction with Machine learning
Source: PLOS Digit Health. 2024 Dec 26;3(12):e0000699. doi: 10.1371/journal.pdig.0000699 (PMC11670925; doi:10.1371/journal.pdig.0000699)
Supplement: S4 Table — (DOCX) [file pdig.0000699.s004.docx]

**S4 Table.** Metafeatures of hospitals.

| **Hospital** | **sample size** | **%ICU** | **%MV** | **%death** | **%missing** | **CV (average)** | **Skewness (average)** | **Skewness (std)** | **Kurtosis (average)** | **Kurtosis (std)** | **Local Training ICU (Yes/No)** | **Local Training MV (Yes/No)** | **Local Training Death (Yes/No)^#^** |
| --- | --- | --- | --- | --- | --- | --- | --- | --- | --- | --- | --- | --- | --- |
| Southeast - 2 | 1500 | 0.699 | 0.443 | 0.358 | 0.221 | 0.570 | 5.129 | 9.968 | 151.566 | 329.113 | 1 | 1 | 1 |
| Southeast - 3 | 449 | 0.675 | 0.365 | 0.236 | 0.106 | 0.427 | 0.893 | 2.630 | 12.686 | 23.390 | 1 | 1 | 1 |
| Southeast - 5 | 124 | 0.218 | 0.161 | 0.137 | 0.227 | 0.880 | 1.983 | 3.099 | 15.042 | 24.688 | 1 | 1 | 1 |
| Southeast - 6 | 56 | 0.429 | 0.232 | 0.232 | 0.148 | 0.929 | 1.160 | 2.385 | 7.307 | 16.096 | 1 | 1 | 1 |
| Northeast - 1 | 1359 | 0.180 | 0.088 | 0.069 | 0.357 | 0.427 | 1.932 | 3.626 | 26.937 | 79.684 | 1 | 1 | 1 |
| Northeast - 2 | 845 | 0.219 | 0.153 | 0.130 | 0.141 | 0.503 | 1.757 | 3.635 | 26.796 | 66.038 | 1 | 1 | 1 |
| Northeast - 3 | 112 | 0.589 | 0.455 | 0.366 | 0.272 | 0.637 | 1.248 | 2.079 | 6.740 | 13.895 | 1 | 1 | 1 |
| Northeast - 4 | 73 | 0.425 | 0.438 | 0.288 | 0.011 | 0.545 | 1.163 | 1.695 | 4.667 | 13.238 | 0 | 0 | 0 |
| MidWest -1 | 539 | 0.954 | 0.570 | 0.609 | 0.089 | 1.075 | 3.429 | 5.247 | 48.468 | 109.485 | 1 | 0 | 0 |
| South - 1 | 456 | 0.300 | 0.186 | 0.103 | 0.396 | 0.619 | 1.669 | 4.856 | 29.378 | 87.206 | 1 | 1 | 1 |
| South - 2 | 148 | 0.142 | 0.074 | 0.068 | 0.018 | 0.395 | 0.914 | 2.107 | 9.538 | 16.205 | 1 | 1 | 1 |
| South - 3 | 91 | 0.505 | 0.374 | 0.374 | 0.212 | 0.779 | 1.282 | 2.367 | 7.860 | 16.972 | 0 | 0 | 0 |
| North - 1 | 247 | 0.304 | 0.186 | 0.154 | 0.242 | 0.357 | 0.551 | 1.598 | 4.473 | 8.137 | 1 | 1 | 0 |
| North - 2 | 47 | 0.723 | 0.574 | 0.340 | 0.076 | 0.413 | 0.695 | 1.166 | 2.427 | 3.715 | 0 | 0 | 0 |
| # Whichmann et al. (2023). | | | | | | | | | | | | | |
