# Supplementary material for: Multicenter comparative analysis of local and aggregated data training strategies in COVID-19 outcome prediction with Machine learning
Source: PLOS Digit Health. 2024 Dec 26;3(12):e0000699. doi: 10.1371/journal.pdig.0000699 (PMC11670925; doi:10.1371/journal.pdig.0000699)
Supplement: S2 Fig — (DOCX) [file pdig.0000699.s006.docx]

| Southeast - 2: Local Training for MV  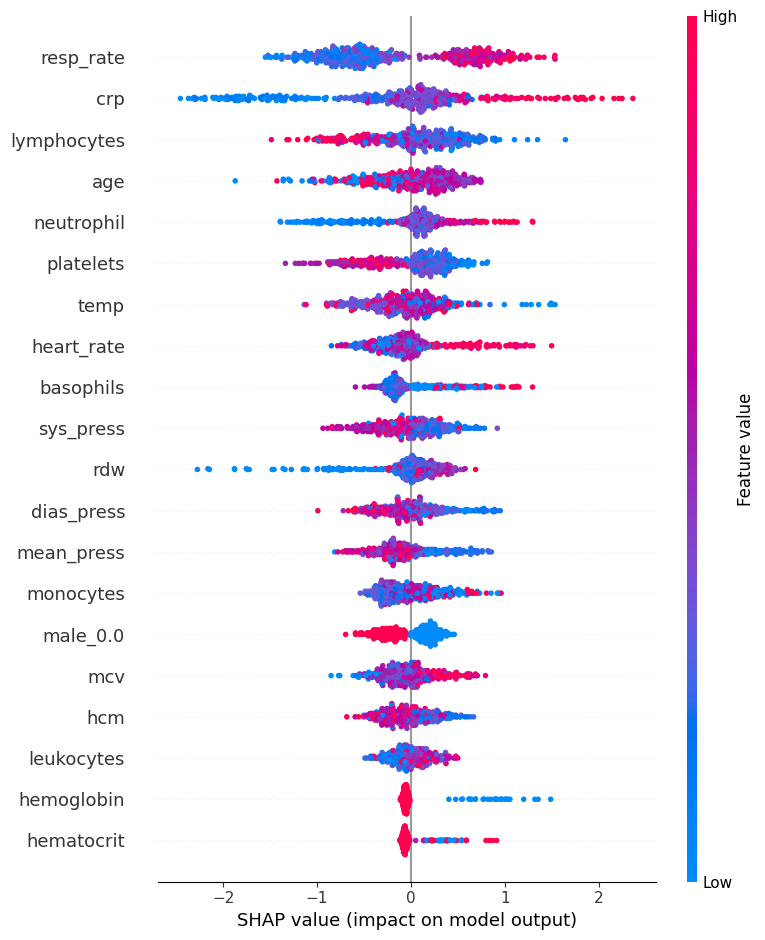 | Southeast - 3: Local Training for MV  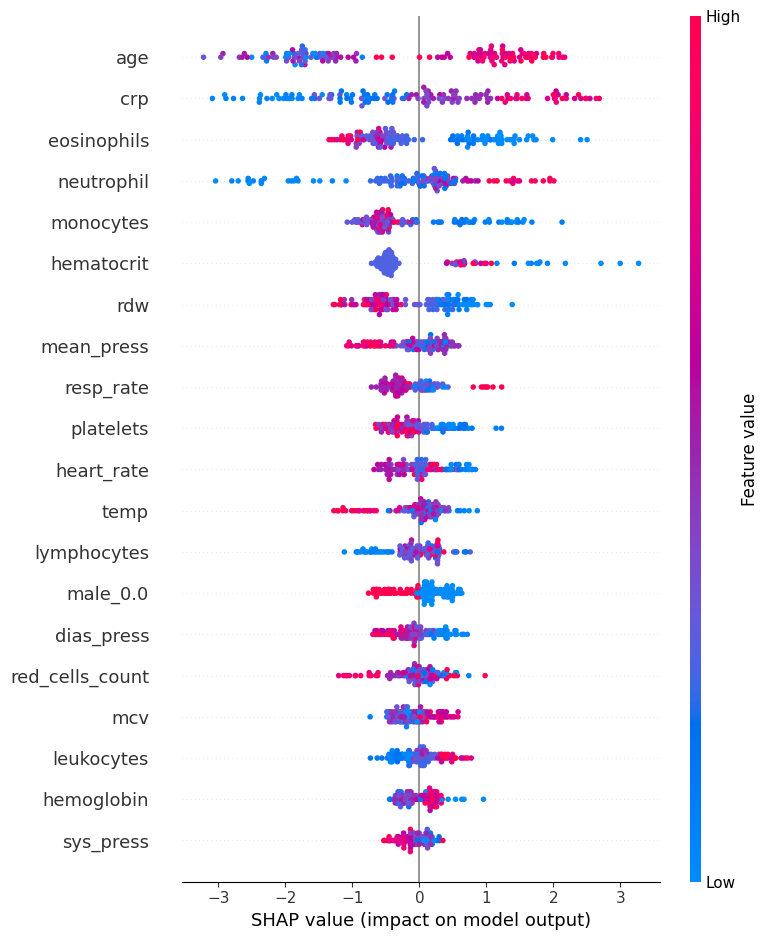 |
| --- | --- |
|  |  |
| Southeast - 5: Local Training for MV  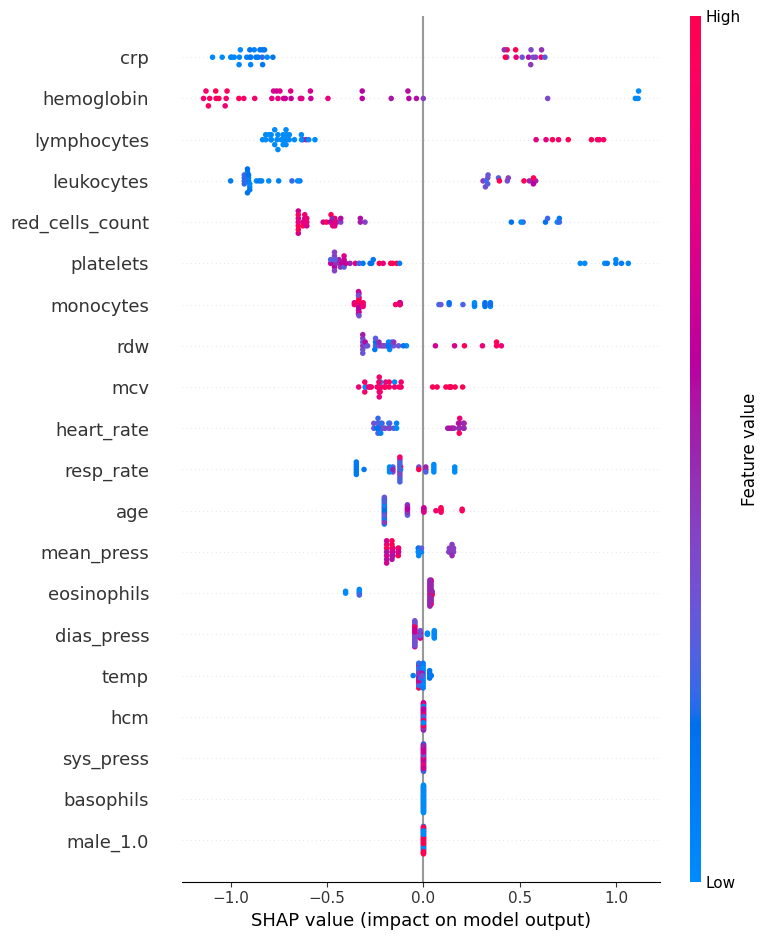 | Southeast - 6: Local Training for MV  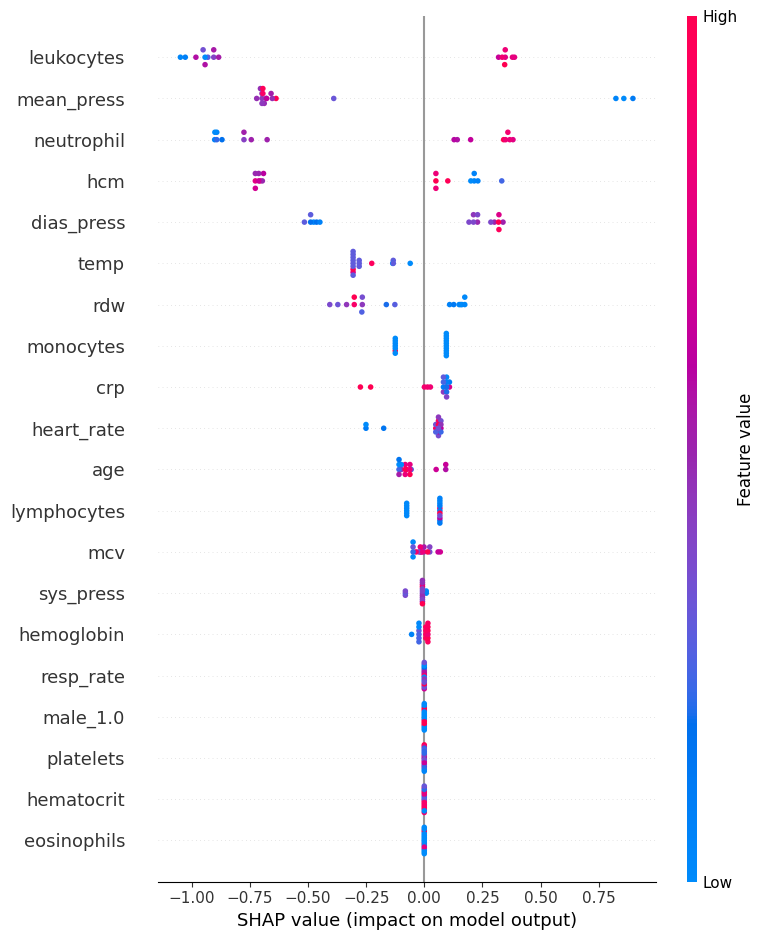 |

**S2A Fig**. The most important predictors according to SHAP values for the best training strategy to predict the mechanical ventilation (MV) outcome for each hospital in Southeast.

| Northeast - 1: Local Training for MV  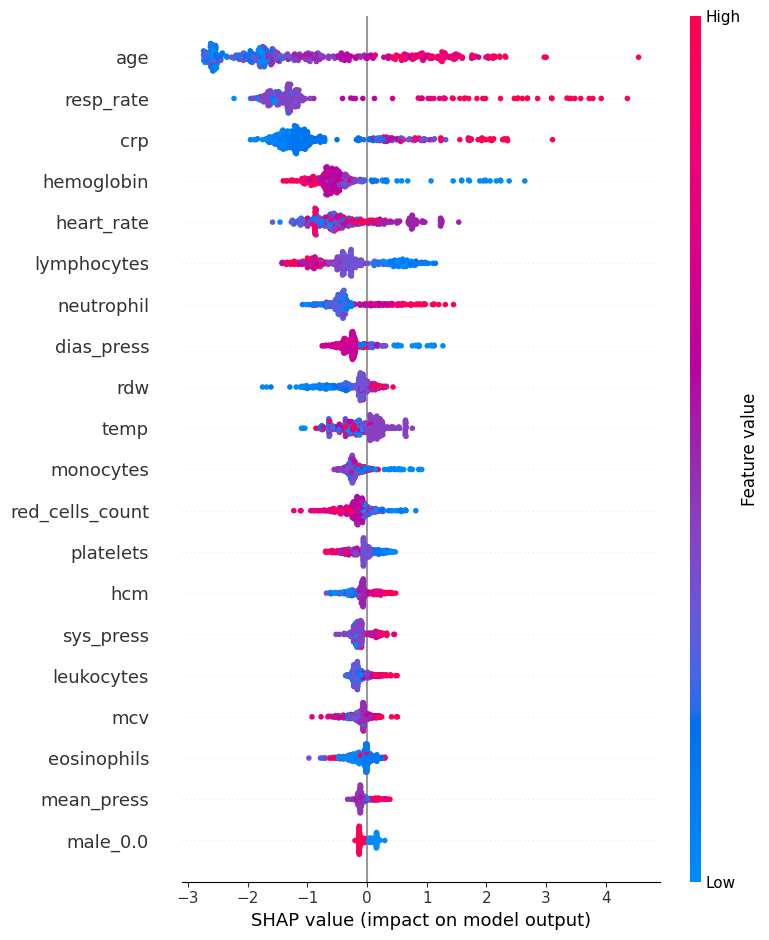 | Northeast - 2: Local Training for MV  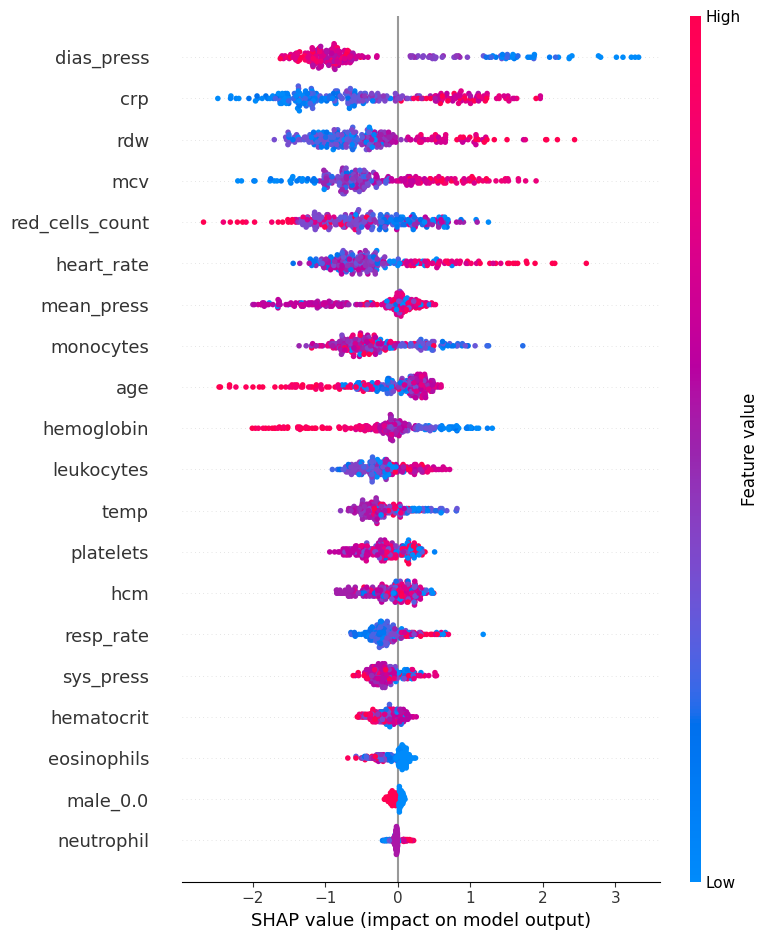 |
| --- | --- |
|  |  |
| Northeast - 3: Local Training for MV  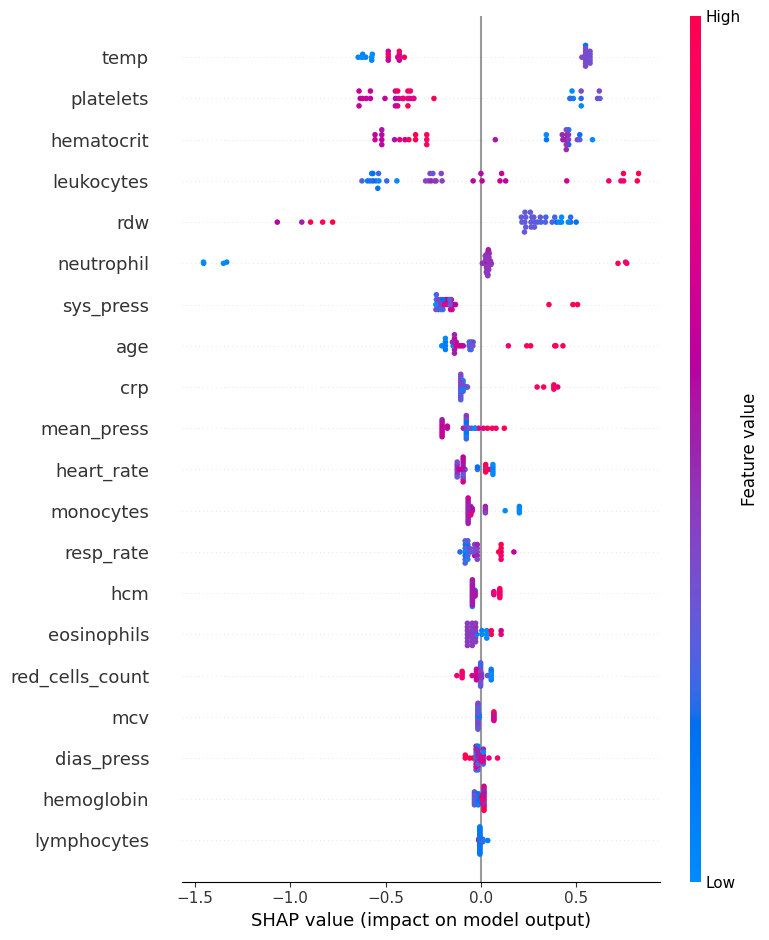 | Northeast - 4: Strategy 7 for MV  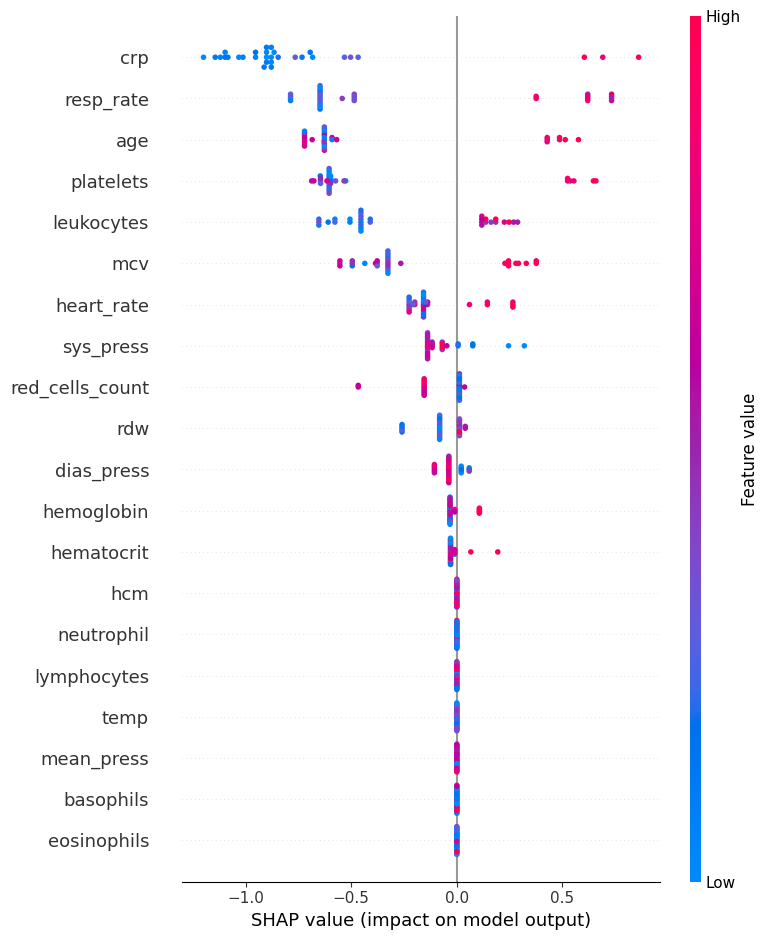 |

**S2B Fig**. The most important predictors according to SHAP values for the best training strategy to predict the mechanical ventilation (MV) outcome for each hospital in Northeast.

| MidWest - 1: Strategy 2 for MV  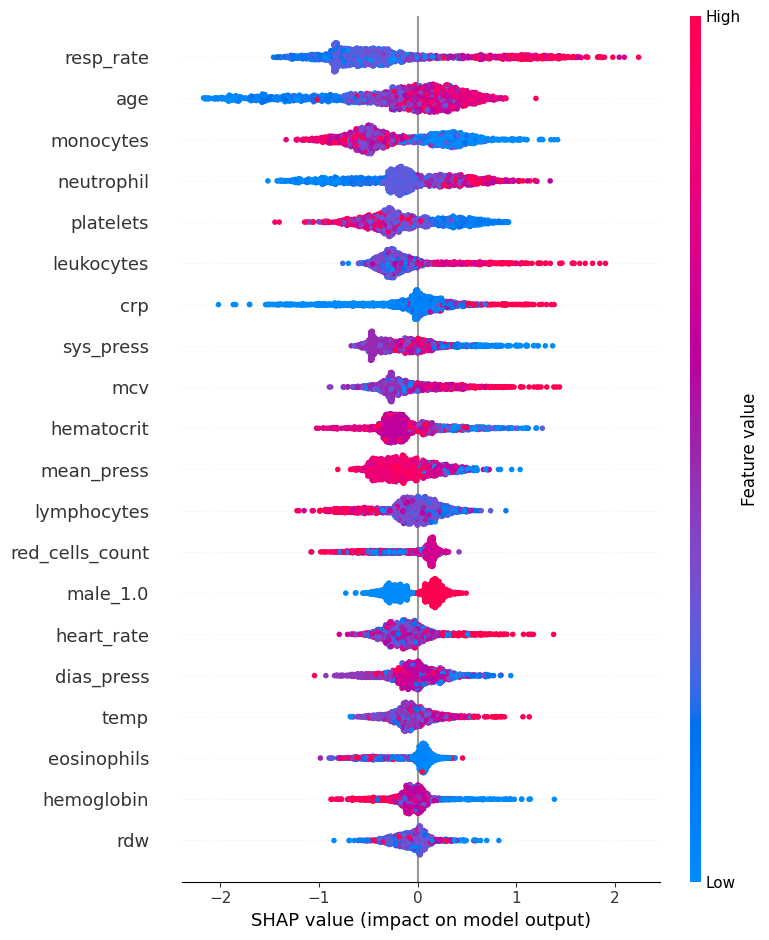 |  |
| --- | --- |

**S2C Fig**. The most important predictors according to SHAP values for the best training strategy to predict the mechanical ventilation (MV) outcome for each hospital in Midwest.

| South - 1: Local Training for MV  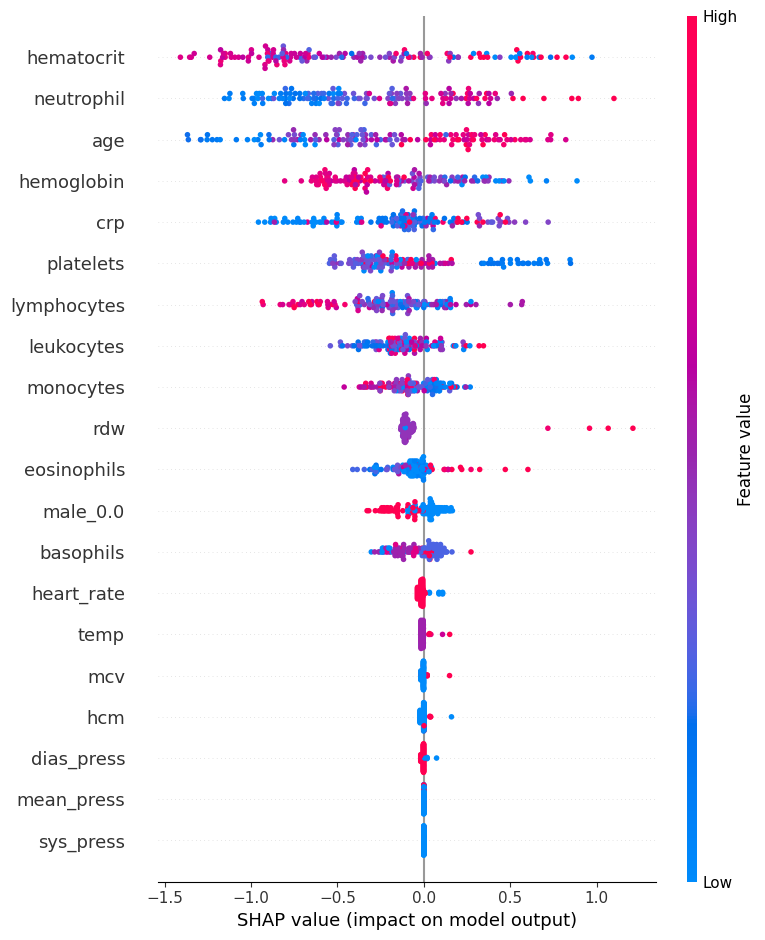 | South - 2: Local Training for MV  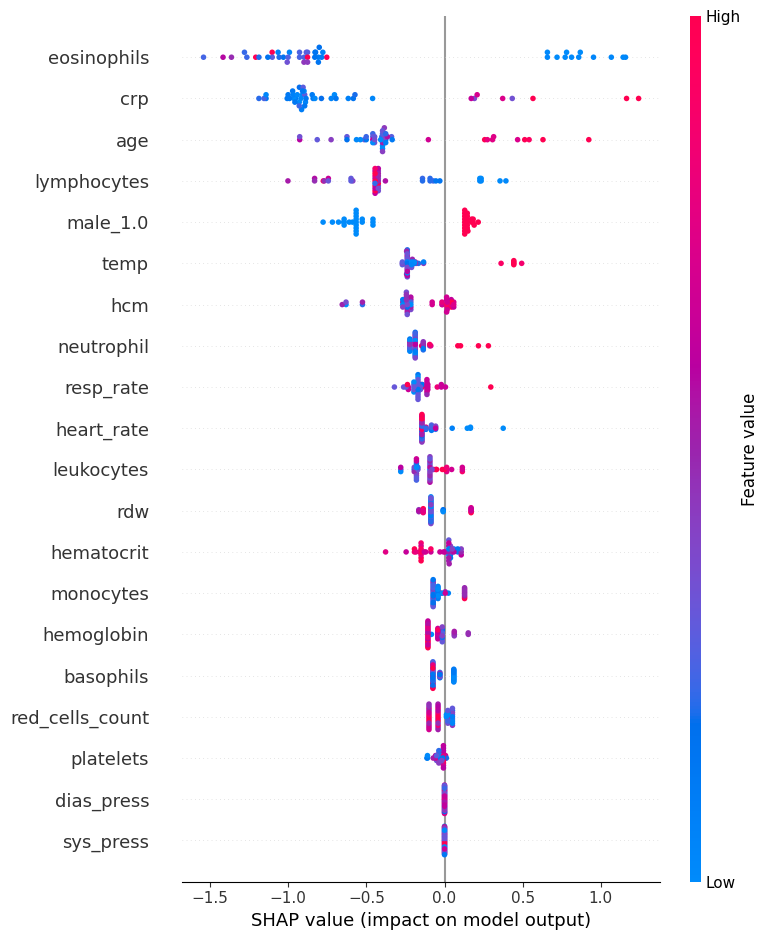 |
| --- | --- |
|  |  |
| South - 3: Strategy 2 for MV  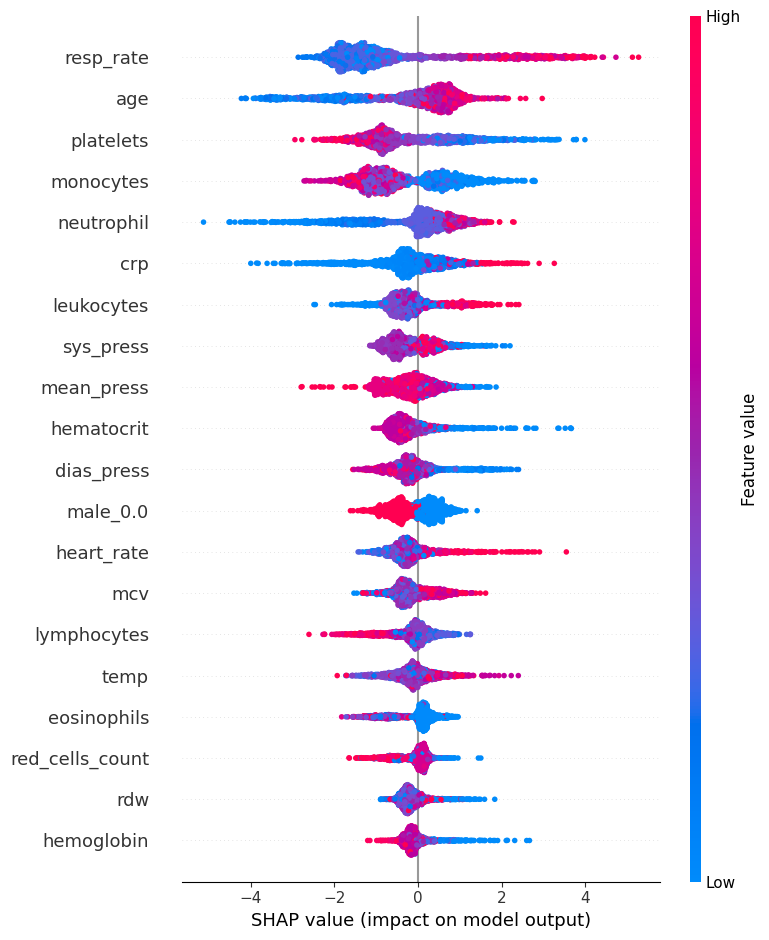 |  |

**S2D Fig**. The most important predictors according to SHAP values for the best training strategy to predict the mechanical ventilation (MV) outcome for each hospital in South.

| North - 1: Local Training for MV  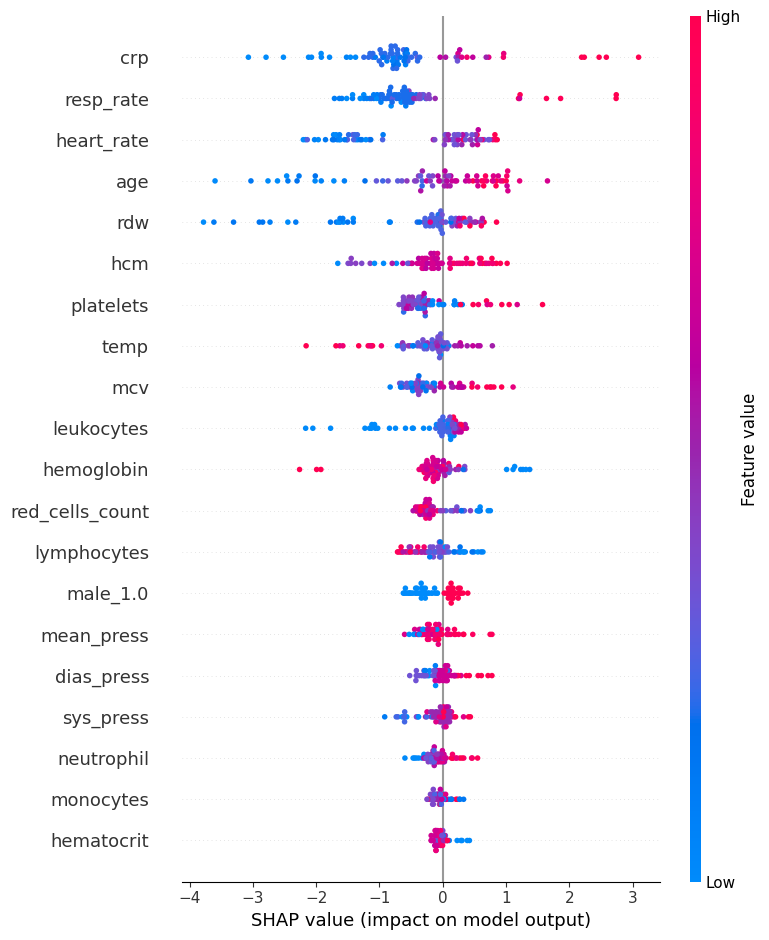 | North - 2: Strategy 2 for MV  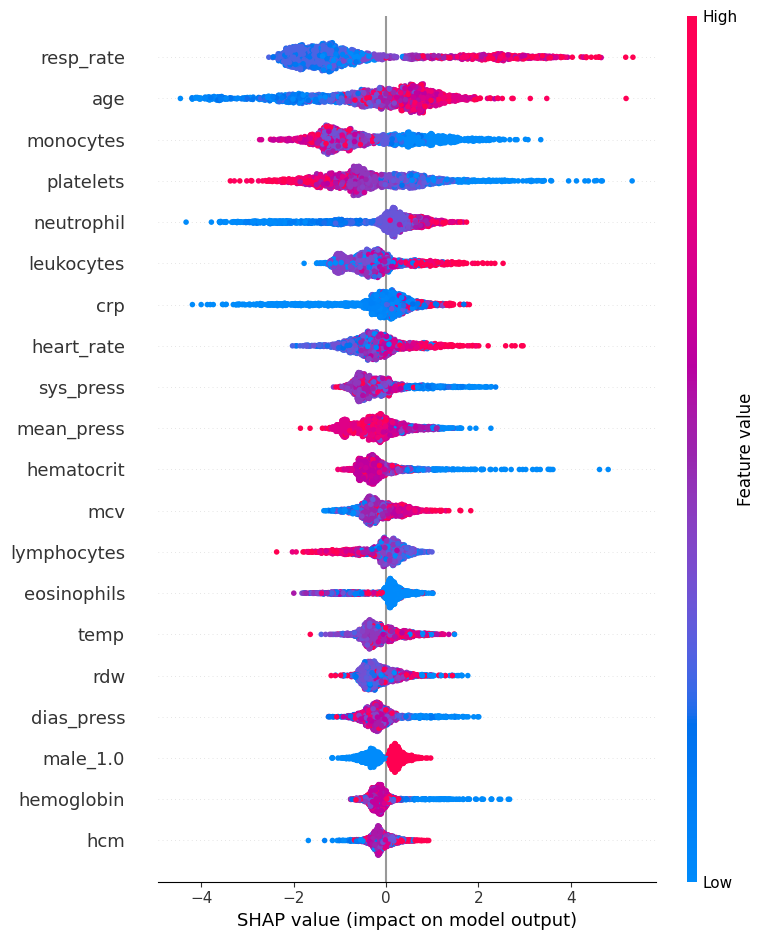 |
| --- | --- |

**S2E Fig**. The most important predictors according to SHAP values for the best training strategy to predict the mechanical ventilation (MV) outcome for each hospital in North.
