# Supplementary material for: Multicenter comparative analysis of local and aggregated data training strategies in COVID-19 outcome prediction with Machine learning
Source: PLOS Digit Health. 2024 Dec 26;3(12):e0000699. doi: 10.1371/journal.pdig.0000699 (PMC11670925; doi:10.1371/journal.pdig.0000699)
Supplement: Attachment — Submitted filename: Rebuttal Letter 08082024.pdf [file pdig.0000699.s007.pdf]

Dear Professor Akhilanand Chaurasia
Editor-in-chief
PLoS Digital Health

Thank you for considering our manuscript “Multicenter comparative analysis of local and aggregated data training strategies in COVID-19 outcome prediction with machine learning” for publication in PLoS Digital Health and for inviting us to revise it. On behalf of all the authors, I am submitting the revised version of the manuscript.

We would like to thank the reviewers for their time and thoughtful feedback. Each of the concerns is addressed in the main manuscript and described point by point below in blue. We hope all comments are addressed sufficiently.

Thank you very much again for your consideration.

Yours sincerely,

The authors

Reviewer #1: As per my observation, this study has significance but couldn't be suitable for publication, since by determining the stays in ICU and ventilation alone, could not assist much in medical significance towards the COVID treatment.

Answer: Thank you for your comment. During the peak of the COVID-19 pandemic, the rapid influx of patients into ICUs and the shortage of ventilators required healthcare professionals to make challenging decisions about resource allocation. While treatments for COVID-19 continue to evolve, machine learning algorithms can play a crucial role by identifying patients at the highest risk of adverse outcomes. This enables the implementation of targeted preventive measures and optimizes the allocation of physical resources within the health system. We have incorporated this information into the Introduction section of the manuscript.

Reviewer #2: Motivation

The paper poses and aims to answer a very important question for digital health: when predicting a patient's outcome from routinely collected medical data, is it better to restrict classifier training to particular hospitals/regions or not. The hypothesis is that variation across hospitals or regions may lead to poorer classifier performance when training data are aggregated.

ANSWER: We completely agree that understanding the effects of regional and hospital variation on classifier performance is crucial for developing more robust and accurate predictive models in healthcare.

Methodology

The paper tests the hypothesis using COVID-19 patient data from 14 Brazilian hospitals. The prediction problem has several input variables (actual number unclear) and 2 output variables (need for intensive care and need for medical ventilation).

ANSWER: Thank you for noticing this. We selected 22 predictors based on their established relevance in clinical assessments for COVID-19, as documented in the literature and in clinical guidelines. We added this information to the Methods section of the manuscript.

The methodology considered eight data aggregation strategies, which include a baseline strategy (total aggregation) and others with varying levels of non-aggregation. Unfortunately this key aspect of the methodology is not well explained and I could not properly understand each strategy. The provided diagram (Fig 1) seemed more of a marketing image rather than explanatory.

ANSWER: In response to your feedback, we have replaced Figure 1 with a new, more detailed figure that better explains the eight data aggregation strategies, including the baseline strategy (total aggregation) and those with varying levels of non-aggregation.

Another methodological uncertainty was the use of oversampling for class rebalancing. The reader was directed to a 200-page text book for explanation. It is not clear what algorithm was used by the authors. It is also not clear why an off-the-shelf state-of-the-are method was not used. E.g., there are implementations of SMOTE (Chawla et al. "SMOTE: synthetic minority over-sampling technique," Journal of artificial intelligence research, 321-357, 2002). Furthermore, many learning algorithms can operate directly with class-reweighting, rather than needing stochastic balancing methods. This was not discussed.

ANSWER: Thank you for your insightful feedback. There are multiple ways to conduct an analysis, and our choice was to standardize the process for all models to ensure consistency. This provides a robust basis for evaluating the impact of different data aggregation strategies. While SMOTE is a well-known method, we opted for an approach that better suited our specific data characteristics and analysis objectives. Preliminary tests indicated that our selected method preserved the original data distribution more effectively. We have included a reference to these tests and their outcomes in the revised version of the manuscript.

I also struggled to clearly understand the learning task given to the machine learning algorithms. The input variables that were considered, discarded and selected was somewhat unclear. Also, the entity of prediction was a patient admission, yet some input variables are measurements which may happen multiple times in an admission. Perhaps there are two or more values for some measurements?

ANSWER: Thank you for noticing these issues. We did not apply a variable selection procedure because our goal was to maintain consistency across all models for comparative purposes. The variables included are widely recognized in clinical research and practice for their relevance in assessing the severity and progression of COVID-19, thus providing a

comprehensive dataset that mirrors real-world clinical assessments. We added a sentence in the Methods section to clarify this point. Regarding the concern about multiple measurements, we have now also specified in the manuscript that we considered only a single measurement for each variable.

A final methodological question exists about the choices of pre-processing to deal with extreme and missing values. The choices seem arbitrary with no justification (or reference to the research question).

ANSWER: Thank you for your question. For handling missing values, we employed the method of multiple imputation using chained equations (MICE), which is a popular strategy to deal with missing data. This method was chosen because it offers a balance between maintaining data integrity and computational efficiency, particularly given the large dataset and the relatively low percentage of missing values. We added this information to the manuscript. Concerning extreme values, we use box-plot to identify extreme values for continuous variables. If the maximum value was at least double the second maximum, it was replaced by missing to avoid non-plausible measures. This strategy helps maintain the overall quality and reliability of the data while addressing potential outliers effectively. We added this information to the manuscript.

Results

The two principle independent variables are "hospital/region" and "aggregation strategy". The principle dependant variable is AUC. Unfortunately, nowhere in the paper is there a table showing AUC for each combination of hospital and strategy. Table 2 is close, but omits the results per strategy. Such results are crucial to the claim of the work. In particular, the reader cannot see if there is a statistical difference in AUC across strategy. Furthermore, the authors do not report statistical tests for the hypothesis that there is a difference.

ANSWER: Thank you for noticing this. We added two new tables (ICU, MV) containing all AUCs for all strategies and all hospitals to the supplementary material (Table S2 and S3). These tables also provide information about the best algorithm in each case. A sentence was added in the results section to refer to these tables. Regarding the AUCs, we followed common practices in the machine learning literature, where an increase in the AUC metric is considered indicative of an improvement in predictive performance.

Other important independent variables mentioned are "training set size" and statistical measures of data variation. These are somewhat systematically treated and reported (Table 3). However, the eight strategies are not made explicit, and conclusions are not clearly tied to these results.

ANSWER: Thank you for highlighting this. We have updated Figure 1 to include detailed explanations of the eight strategies. Additionally, we have revised the Methods, Results, and Discussion sections to clarify the role of metafeatures, such as training set size and statistical measures of data variation, and how they relate to the eight strategies. These

revisions aim to better connect the conclusions to the reported results and provide a clearer understanding of the impact of these variables.

The results of Shapley value analysis, while interesting, do not seem to directly relate to the posed research question. The relevance of Shapley values to the research question could be made clear (or the analysis omitted).

ANSWER: Thank you for your observation. Shapley values were included to assess the contributions and potential overlaps of the main predictors used in the best model for each hospital. We have added clarifying sentences to the Discussion section to better explain the relevance of this analysis to the research question.

Conclusions

The authors seem to claim:
(1) "a consistent tendency of better predictive performance for severe outcomes in hospitals with disaggregated data"
(2) "the use of data from other hospitals to train algorithms for predictive purposes could increase data noise due to heterogeneity in hospital practices"
(3) hospitals with small training set sizes may benefit from data aggregation (see below),
(4) their "findings offer practical insights".

Claim (1) may be true and the authors may have collected the necessary data, however, the statistical validity of the claim is not established.

ANSWER: Thank you for pointing this out. We acknowledge that Claim (1) is not completely established from the results, even if we have observed an overall tendency for better predictive performance with disaggregated data. We have now toned down these claims, and clearly stated these limitations of our current analysis.

The results supporting claim (2) may be present in Table 3, however the authors could help the reader to see the connection.

ANSWER: We added a sentence this information to the Discussion to better explain what we meant by “data noise due to heterogeneity in hospital practices”.

Claim (3) was difficult to interpret (lines 207 to 211 on page 12). It would really help if the authors could clarify what they are claiming here, and make a clear connection to the presented results.

ANSWER: Thank you for pointing this out. In Claim (3), we intended to convey that the hospitals benefiting from data aggregation were not concentrated in any specific region; rather, they frequently shared a common characteristic of having small sample sizes. We

have rephrased this section for greater clarity and to better connect it to the presented results.

I am not convinced that claim (4) is established. In particular, I can't see where authors have explained to the reader what important consideration there are when deciding to aggregate data or not. Nor have they provided guidance on what may be appropriate aggregation methods.

ANSWER: Based on our findings, we observed that only hospitals with small sample sizes benefited from aggregated data. This observation provides valuable practical insights, helping future studies make informed decisions about the benefits of data aggregation. To highlight this, we added a sentence at the end of the Discussion emphasizing the importance of considering sample size when deciding whether to aggregate data and suggesting appropriate aggregation methods.

The authors finish with, "Our findings highlight the need for careful consideration when combining data from different regions [...]." However, they do not state what those considerations are.

ANSWER: Thank you for noticing this. We added a sentence to the end of discussion to address this point.

The authors also state, "These predictions assist clinicians in making informed decisions about treatment and resource allocation [...]." However, the cited work does not seem to make a good case for using such predictive analytics (like that presented by the authors) in clinical decision making.

Thank you for your feedback. We agree; the cited work was incorrectly placed in the text. We have moved the citation to the previous sentence. Our intention in this case iwas to emphasize the importance of predicting outcomes related to COVID-19, such as ICU admissions and the need for mechanical ventilation (MV), and to highlight the potential of machine learning as a tool for these predictions.

Language

The prose of the submission I found was often ambiguous and lacked the precision expected of a scientific publication. Beyond me failing to understand the aggregation strategies, I also could not understand what was "the" RT-PCR exam (were there one or many, when did it happen, how was it used? what was the patient workflow?). There is much mention of "the reference hospital" but it was not clear which one was the reference hospital or what its purpose was in the method. Another minor example is that the authors state, "This study aims to compare data aggregation strategies [...] to predict COVID-19 outcomes such as [...] ICU and [...] MV." Why say "such as" when actually, it was "specifically" those two outcomes?

ANSWER: Thank you for your comments. We have addressed these points in the introduction and made several changes throughout the text to improve clarity. Specifically, we clarified the use of the RT-PCR exams, detailed the patient workflow, and identified the reference hospital and its role in the study. Additionally, we replaced "such as" with "specifically" to accurately reflect that the study focused on predicting ICU admissions and mechanical ventilation (MV) outcomes. Figure 1 has been updated to further clarify these aspects.

There seem to be a few typos when referring to figures and tables. E.g., line 78 on page 4, line 180 on page 10, and elsewhere.

ANSWER: We corrected the issues according to the journal’s "instructions to authors”.

When describing their work, the authors make a deal about the fact that data were extracted from 18 hospitals, but only data from 14 hospitals was analysed. This does not seem to be a big deal, but the reader is reminded of this fact multiple times from some reason.

ANSWER: Thank you for your feedback. To improve clarity, we have rephrased the relevant sentences to concisely explain this point about hospital exclusion.

Data Availability

Finally, a comment regarding data availability. The authors claim they cannot release data due to "Brazilian data protection law". However, it is not clear what aspect of the cited law prevents release of deidentified records yet permit release given a "reasonable request". Furthermore, it is not clear what constitutes a "reasonable request".

ANSWER: Thank you for your comment regarding data availability. We understand the need for clarity on this matter. The Brazilian General Data Protection Law (LGPD) restricts the public release of deidentified records to protect individuals' privacy. However, the law allows for data sharing under specific conditions that ensure privacy is maintained, which is why we mention that data can be released upon a "reasonable request." A "reasonable request" typically refers to a request made for legitimate research purposes, where the requester can demonstrate a clear plan for maintaining data confidentiality and ethical standards. We will provide additional details in the manuscript to clarify these points.

Reviewer #3: Although the prevalance of COVID 19 has abrupted and the assesssed technology doesn't provide good results when large data set was used, the study could be quite helpful for the other future researchers who will work on the stated technology, It would be great insight for them, by referencing the present conducted research. This also increases the scope for modifications in the assessed technology for the long run, in order to tackle the bottlenecks of the present study.

ANSWER: We thank Reviewer #3 for the positive feedback and insightful comments. We appreciate the recognition of the potential long-term value of our study, despite the limitations observed with large datasets. We agree that our findings can serve as a valuable reference for future researchers working with this technology, providing insights and opportunities for improvements. By addressing the bottlenecks identified in our study, future research can further enhance the effectiveness and applicability of machine learning algorithms.
