# Supplementary material for: Multicenter comparative analysis of local and aggregated data training strategies in COVID-19 outcome prediction with Machine learning
Source: PLOS Digit Health. 2024 Dec 26;3(12):e0000699. doi: 10.1371/journal.pdig.0000699 (PMC11670925; doi:10.1371/journal.pdig.0000699)
Supplement: Attachment — Submitted filename: Reviewer Letter 28102024.pdf [file pdig.0000699.s008.pdf]

Dear Dhiya Al-Jumeily OBE
Section Editor
PLoS Digital Health

Thank you for considering our manuscript “Multicenter comparative analysis of local and aggregated data training strategies in COVID-19 outcome prediction with machine learning” for publication in PLoS Digital Health and for inviting us to revise it. On behalf of all the authors, I am submitting the revised version of the manuscript.

We want to thank reviewer 2 for the additional review. We hope that all comments are satisfactorily addressed.

Again, thank you very much for your consideration.

Yours sincerely,

The authors

Reviewer #2: Overall

The authors have done an excellent job of addressing most of the feedback provided in my initial review. However, some outstanding issues still remain that may be easy to resolve.

The authors have improved the justification for performing oversampling. However, it is still not clear to me which oversampling technique was used. The provided reference is a text book. Perhaps consider directing the reader to a particular page within the text that describes the method used.

ANSWER: Thank you for your observation. The technique used was random oversampling. We added this information to the manuscript and replaced the reference of the book with a reference of a paper that addresses the imbalance issue and applies the random oversampling method as one of the proposed methods to deal with it.

11.Batista GEAPA, Prati RC, & Monard MC. A Study of the Behavior of Several Methods for Balancing Machine Learning Training Data. SIGKDD Explorations, 2004; 6(1): 20-29

It is still not clear to me if source variables were removed. The following two quotes seem to contradict.
1) "The 22 variables were kept in all models to maintain consistency across models for comparative purposes." [p5 line 86].
2) "Variables exhibiting a correlation coefficient exceeding 0.90 were removed. Additionally, variables with over 90% missing values were also excluded." [p5 line 93]

ANSWER: Thank you for bringing this to our attention. We have refined the first sentence for clarity. Initially, all models included 22 variables. However, we later addressed issues related to collinearity and a high proportion of missing data. As a result, this analysis led to adjustments in each hospital's predictors list.

The description of the methodology is much improved (Table 1 really helps). However, I still struggle to fully understand it. After much analysis of the revised text I think I may understand it. I have summarised my understanding below. If this understanding is correct, I offer it as a possible framing for improving the description of the methodology.

ANSWER: Thank you for your feedback and suggestions. They have been instrumental in revising the description of this methodology which is challenging to explain. We have incorporated the suggestions, and we believe that the new explanation is now clearer.

I cannot find a statistical analysis of the primary hypotheses, namely that the "Local Training is better" and "Local Training is not better for small sample sizes." (I apologise if I have missed it.) The authors have provided all the data needed to perform such an analyses - which is excellent - but an analysis seems missing. I have performed my own analysis (below) and the results are favourable for the authors' claim. If this analysis is useful for the authors, I offer it as a possible template for their own analysis.

ANSWER: Thank you for your comments and suggestions. We have clarified the hypothesis that hospitals with small sample sizes would benefit more from data aggregation. Additionally, we have included the binomial tests and confidence intervals in the manuscript as suggested.

My Understanding of the Methodology

There are 14 hospitals (each is within 1 of 5 regions).
There are 8 strategies. For each strategy there are 14 trials [my terminology], where each trial is associated with one of the 14 hospitals, designated as "the reference hospital" for that trial.
Thus there are 8 x 14 = 112 possible strategy-hospital combinations. However, 2 combinations were not possible, explained below, i.e., there are only 110 strategy-hospital combinations. Each strategy-hospital is applied to 1 of 3 learners. There are two possible outcome variables. Thus there are 110 x 3 x 2 = 660 strategy-hospital-learner-outcome combinations all together.
For each hospital, the cases are partitioned so a [I assume random] 30% of cases are reserved for testing and the remaining 70% are used for training.
It is not clear if the train/test partitions were re-randomised or kept constant over the methodology. Specifically, are the same cases used for training/testing for each strategy-learner-outcome combination, or is data randomly repartitioned for each different strategy, for each different learner, for each outcome variable?

For each strategy-hospital-learner-outcome combination a classifier is trained. The training set is constructed according to a strategy and is relative to the trial's reference hospital. Then the classifier is applied to the reference hospital's testing data.
Strategy 1: train on the reference hospital.
Strategy 2: train on all hospitals except the reference hospital.
Strategy 3: train on hospitals in the same region except the reference hospital.
Strategy 4: train on hospitals _not_ in the same region as the reference hospital.
Strategy 5: train on all hospitals, including the reference hospital.
Strategy 6: train on the reference hospital + a sample of the same size from all other hospitals.
Strategy 7: train on the reference hospital + a sample of the same size from other hospitals of the same region.
Strategy 8: train on the reference hospital + a sample of the same size from other hospitals _not_ in the same region as the reference hospital.
The Midwest region contained only one hospital, thus strategies 3 & 7 had no trial with that hospital as a reference.
For each strategy-hospital-learner-outcome combination, AUC was calculated. The classifier with the highest AUC was selected from each strategy-hospital-outcome.
The best strategy per hospital-outcome was found (modulo the two excluded strategy-hospital combinations). These 14 x 2 = 28 results are shown in Table 3.

My Statistical Analysis

Hypothesis: local training is better.
Null hypothesis: binomial distribution, 0.5 chance.
(ICU experiment)
Data: 11 of 14 (0.7857).
99% CI (Clopper-Pearson exact): 0.4108 -- 0.9743
p-value = 0.0287
(MV experiment)
Data: 10 of 14 (0.7143).
99% CI (Clopper-Pearson exact): 0.3421 -- 0.9474
p-value = 0.0898
(ICU + MV)
Data: 11 + 10 of 14 + 14, i.e. 21 of 28 hospital-outcome trials (0.7500).
99% CI (Clopper-Pearson exact): 0.4924 -- 0.9214
p-value < 0.00000001
(ICU + MV, with sample size >= 112, i.e., excluding small sample size)
Data: 10 + 9 of 10 + 10, i.e. 19 of 20 hospital-outcome trials (0.9500).
99% CI (Clopper-Pearson exact): 0.6829 -- 0.9997
p-value = 0.00002
